# Using examination performance data and focus groups to inform teaching – a case study from final year students of veterinary medicine

**DOI:** 10.1186/s13620-020-0155-3

**Published:** 2020-01-10

**Authors:** Conor G. McAloon, Emmet Kelly, Sue Rackard, Catherine McAloon, Marijke Beltman, Luke O’Grady, Lorenzo Viora, Mark Crowe, Michael L. Doherty, Eoin G. Ryan

**Affiliations:** 1 School of Veterinary Medicine, University College Dublin, Belfield, Dublin, D04 W6F6 Ireland; 2 Scottish Centre for Production Animal Health and Food Safety, University of Glasgow, School of Veterinary Medicine, Bearsden Road, Glasgow, G61 1QH UK

**Keywords:** “Dairy” “focus groups” “teaching evaluations”

## Abstract

**Background:**

Student feedback has played an important role in the maintenance of quality and standards in higher education. Perhaps the most commonly used method to capture feedback is a series of questions or statements where students indicate their degree of satisfaction or agreement. Focus groups offer an alternative means of capturing ‘richer’ qualitative data relating to students’ thoughts on course structure. Aside from student evaluations, student examination performance has been used as a method to evaluate the efficacy of curriculum changes at programme level. However, this data is utilised less so at a ‘finer detail’ level to identify specific issues with the delivery of teaching.

**Case presentation:**

The purpose of this report was to outline the approach taken using qualitative and quantitative data to identify problems with a specific area of teaching, inform a new teaching approach and to assess the impact of those changes. Following quantitative and qualitative analysis, a practical class on dairy herd fertility performance was highlighted as an area for improvement. After the introduction of the newly formatted practical class with a greater focus on self-directed learning, there was a significant increase in the average score (*p* < 0.001) and a decrease in the proportion of students failing (p < 0.001) the question that assessed the analysis of dairy herd fertility data. In addition, the R-squared value between students’ performance in the fertility question and their performance in the overall examination increased from 0.06 to 0.11.

**Conclusions:**

The combination of qualitative focus group data and quantitative analysis of examination performance data represent robust methods for identifying problems associated with specific aspects of veterinary teaching.

## Introduction

Student feedback has played an important role in the maintenance of quality and standards in higher education [1]. Previous authors have outlined several reasons for undertaking student evaluations of teaching effectiveness, including for example, for the benefit of other students deciding on course selection [2, 3]. However, probably the most common reason for undertaking student evaluation is for ‘diagnostic’ reasons to identify potential issues and solutions to improve the quality of course teaching [3].

A range of methods are available to capture student feedback. Perhaps the most commonly used method is a series of questions or statements where students indicate their degree of satisfaction or agreement [3]. However, a potential criticism of this approach is that these survey-type instruments in general have a low response rate and are potentially subject to a range of student biases [4, 5]. Furthermore, surveys in general do not facilitate the capture of “deeper” data relating to the thoughts, feelings and experiences of the participants [6]. Such data may be obtained through qualitative approaches.

Focus groups offer a means of capturing qualitative data relating to students’ thoughts on course structure. They traditionally consist of structured discussions of defined duration and are particularly useful in identifying problems and potential solutions in course structures and teaching methods [7].

Aside from student evaluations, student performance in examinations may be used as a means of evaluating teaching effectiveness. At a programme level, the efficacy of curricula have been assessed by evaluating student performance in board examinations [7]. However, there are less examples of the use of examination data to evaluate teaching at the ‘micro’ level, i.e. with regard to the teaching of very specific topics within the programme. Although examination component or item analyses are often carried out, the inferences from these analyses are often made at the level of the question or at the level of the examination. For example, poor performance on a particular question may often be attributed to difficulty of the question, whilst a poor discriminatory power of a question may be related to the quality of that question [8]. However, consistently poor performance on similar questions on the same topic may indicate poor efficacy of teaching methods or a deficiency in the curriculum, rather than difficulty of the question [9].

The purpose of this report is to outline how both qualitative and quantitative data may be collected and used to identify problems with a specific area of teaching, inform a new teaching approach and to assess the impact of those changes.

## Case presentation

Final year teaching in the School of Veterinary Medicine, University College Dublin (UCD) is a lecture-free, rotation-based course covering a number of modules including farm animal clinical studies (FACS), equine medicine, large and small animal surgery, small animal medicine, diagnostic imaging, anaesthesia and paraclinical studies. The teaching of FACS is delivered over two rotations; one in first semester lasting 3 weeks and a second rotation in semester 2 lasting 2 weeks. Farm animal practice in Ireland is highly seasonal with the bulk of clinical work occurring in spring, reflecting the high proportion of spring-calving herds in Ireland. Clinical exposure in the FACS rotation reflects this seasonality with the majority of individual medical and surgical cases presenting in semester 2 and a greater proportion of practical classes and tutorials based around herd health management taking place in semester 1.

### Focus group - participant recruitment

Students were recruited using convenience sampling from the student led UCD Farm Animal Veterinary Society. Students from this society were sought in order to improve engagement with the feedback process. In addition, it was also envisaged that students intending to pursue a career in farm animal veterinary would have given more thought as to how teaching within the module prepared them for life in practice as a farm animal veterinarian.

One final year student who was known to be a member of the society was approached to contact student colleagues who intended to pursue a career in farm animal veterinary practice after qualifying, requesting that they attend a focus group designed to obtain feedback on the module. Students who had already completed both rotations were preferentially selected for providing feedback so that they could give feedback on the entire module. The session took place in the evening and students were provided with food (delivery pizza) for their involvement in the group. The session was recorded on a dictaphone for later transcription after first receiving consent from all the participants. All data were anonymised at transcription. The process was granted ethical exemption from UCD Human Research Ethics Committee (LS-E-19-145-McAloon).

### Focus group - approach

This session was designed as a facilitated focus group. Before the session, the instructors teaching on the FACS module broke the core teaching activities down into various methods of delivery (student exercises/presentations, clinical activity/teaching, tutorials and practical classes), as well the aspects of assessment and feedback. All components under each section were listed to serve as a reminder of what teaching had been provided throughout the duration of each rotation. Summary sheets for each teaching method were printed out prior to the session. Each teaching method was addressed in turn and the same questions were asked; *“What worked well?”, “What didn’t work well?”, “Do you think this material could have been delivered in a better manner?”* and *“How would you improve this part of the module?”*. The session was conducted with minimal prompting from the facilitator. In addition to recording the session the facilitator kept notes of particular areas of interest for further probing.

After transcription, the first author initially read through the data to become familiar with the material. The data were examined with a focus on the objective of the study, (i.e. evaluation of the teaching methods in the module). Codes were initially established for each of the points made during the discussion and these subsequently developed into themes.

### Examination data analysis

The examination for the FACS module includes Single Best Answer Questions as well as a series of structured short answer written questions. Data were extracted for each students’ performance in each question.

After data were anonymised, indicators of item difficulty i.e. the average score for each question, as well as the proportion of students failing (scoring less than 50%) were calculated for each question in each year. In addition, the Pearson’s correlation coefficient between the students’ score in each question, versus their performance in the rest of the examination (i.e. excluding their score in the question being assessed) was calculated. This figure gives an indication of how well an item discriminates between good and bad performing students. Squaring this (correlation coefficient) gives the coefficient of variation (R-squared). This coefficient of variation represents the proportion of the variation in one variable that is explained by the correlated variable. In the context of this study, the R-squared figure answers the question: *How much of the variation in item test score is explained by the students’ performance in the rest of the examination?* Ideally, this question would infer directly on the students’ ability or ‘true test score’, that is, how much of the variation in a question is explained by the ‘true test score’. However, since this ‘true test score’ is unobserved, the R-squared value between each question and the score attained in the rest of the examination (minus that question being assessed) can be used as an estimate of the relationship between the individual question score and candidates’ unobserved ‘true score’ using Classical Test Theory [10].

Finally, a two-sided t-test statistic for two sample means was used to test the null hypothesis that the means did not differ between 2 years whilst the proportion of students failing was compared across each year using the Chi-squared statistic. For both sets of comparisons, all two-way comparisons between each of the 3 years were compared. All data analysis was performed in R-studio [11].

## Results

### Adaptation of teaching methodology

Following the completion of the review process in 2017–18, one topic (dairy herd-level fertility analysis) was highlighted for tutorial redesign due to poor student performance in these questions and direct student feedback identifying important deficiencies in how the material was being delivered. Dairy herd-level fertility analysis involves the calculation and interpretation of simple fertility Key Performance Indicators (KPIs) for a dairy herd. These skills map directly to Day One competencies for farm animal veterinary graduates. Prior to redesign, students had been taught in a single tutorial where students received a short lecture on dairy herd-level fertility analysis followed by a brief practical session. During this practical session, they were divided into small groups and presented with raw fertility data in Microsoft Excel and asked to calculate and interpret basic KPIs.

Following this analysis, the class was redesigned for the 2018–2019 academic year. A student-led problem-based learning approach was identified as the preferred teaching methodology. Students were organised into groups of 6 or 7 and each group was presented with a dairy herd fertility problem based on a real scenario. The problem included a brief history and some raw fertility data in an Excel worksheet. Each group was asked to calculate some simple herd KPIs from the raw data and make some interpretations and recommendations regarding fertility management in each herd with regard to the presenting problem. Over the course of the 3-week rotation, the students were given the time and opportunity to examine the data in detail while working together within the group, and to source materials to help with the calculations, interpretations and recommendations. If required they were also allowed to contact the facilitator for some direction. Each group was then asked to present the material to the rest of the rotation groups and their findings were discussed with the facilitator and other participating groups.

### Focus group

The purpose of the focus group was to capture data on the teaching methods across all components of the module. Seven participants were successfully recruited, all of which were members of the Farm Animal Veterinary Society. The themes identified followed the general structure of the topics for discussion in the focus group that is the teaching methods used in the module. In general students were positive about the majority of the teaching methods and taught material. However, across all of the categories of teaching methods points were highlighted for improvement. These ranged from the timing of student presentations:

*“No reason why can’t run this (nutrition presentation) in the first or second week… (we’d) definitely have enough time”.*

To a greater desire to undertake self-directed learning:

*“I think we’ve spent enough time at this stage like sitting in lecture halls and chatting and looking at powerpoints and they are great like. And definitely coming up to exams I’m going to be reviewing you know (tutorials and lectures) and everything. But you know as the lads were saying it’s probably no harm for us to go away and look into these things ourselves.”*

When discussing fertility teaching students highlighted the benefit of a practical class on rectal palpation. However, a follow up practical class on herd-level fertility analysis was highlighted as one in which the students found difficult and felt confused regarding terminology and the calculation of fertility KPIs, although they also felt that the class was important:

*“I think the fertility performance analysis confused a lot of people. (but) I don’t … think it needs to be dropped…”.*

In particular, students felt there was perhaps insufficient time to allow them to grasp all of the material delivered in a single tutorial:

*“We got to a certain level and then it (ended)”.*

Students expanded that following the class, there was confusion over the different KPIs and how they were calculated.

### Examination performance data

From the academic years 2016–17 to 2018–19, a short structured written answer question was set on basic herd-level fertility data analysis. Whilst the specifics of the question varied each year, in general the same competencies were assessed. Students were provided with a table summarising calving spread in a seasonal dairy farm (first calving date, median calving date, last calving date), conception rate, submission rates and percentage pregnant at the end of the breeding season. From this table, they were asked to comment on the performance of the herd. Next, a basic table was provided to allow students manually calculate differences in conception rates between different groups of individuals either according to parity; artificial insemination (AI) versus natural service; or farmer performed AI versus technician performed AI. Students were asked to calculate the conception rate for each of these groups and to comment on differences observed between this KPI across the groups.

Table 1 shows the performance of students across this question type from 2017 to 2019. The average score and proportion of students failing the fertility question in 2016–17 was 6.7 and 20% respectively. The comparable figures for 2017–18 were 6.2 and 26%. There was no statistically significant difference between the figures across both of these years. The R-squared values in 2016–17 and 2017–18 were 0.05 and 0.04 respectively.

**Table 1:** Performance over 3 years, of students on a question dealing with analysis of dairy herd fertility. Figures within the same column but with different superscripts are significantly different (*p* < 0.05)

Year	Average Score	Proportion failing	R-squared
2016–17	6.7^a^	20%^a^	0.05
2017–18	6.2^a^	26%^a^	0.04
2018–19	7.5^b^	6%^b^	0.11

After the introduction of the newly formatted practical class, there was a significant increase in the average score (*p* < 0.001) and a decrease in the proportion of students failing (p < 0.001). In addition, the R-squared value increased to 0.11.

## Discussion

This report outlines an approach taken to identify a specific issue with teaching efficacy within a course and to assess the value of the changes made to the teaching method introduced.

The use of examination results to assess the efficacy of curriculum changes is well documented at the programme or curriculum level [12]. However, there is less documented use of using these approaches at what might be considered the ‘micro’ level, i.e. to assess specific aspects of teaching within a module of a specific programme.

After using combined qualitative approach along with a detailed quantitative analysis of the performance of specific examination questions, we identified a specific issue in the delivery of teaching of the analysis of dairy herd fertility data.

Quantitative data analysis showed a relatively high difficulty in fertility questions each year as demonstrated by the mean question scores and the proportion of students failing. In addition, the proportion of the variation in question score explained by the students’ overall ability was relatively low. Taken after a single examination and on a single year, such findings could indicate a poorly designed question. However, longitudinal analysis supported an alternative inference, i.e. that there was a deficiency in the delivery of the relevant teaching material.

The initial approach taken to deliver teaching with this aspect of the module was in the form of a practical class, whereby students followed an instructor on the calculation of the relevant fertility KPIs. In contrast, the qualitative data gathered from the focus group discussions highlighted a preference for students to undertake greater self-directed learning. This data was used to direct the renewed approach which assigned the students a greater responsibility in the learning process. Students were given real-life herd fertility problems and asked to calculate basic KPIs, interpret the findings and come with recommendations for the farmer. Rather than just following instructions on the calculation of fertility KPIs, students were now given time to reflect on the problem; think about the summary data that would be most useful to calculate; source information on how to calculate and interpret indices with respect to targets for their assigned production system; interpret the findings in light of the problem faced and come up with practical solutions to the farmer that were specific to their inferences from the data.

After redesign of the delivery of teaching in this area within the final year rotation, we observed a statistically significant increase in the mean score for the question, as well as a significant decrease in the proportion of students failing the question. These findings reflect increasing movement in medical education from teacher- to student-centred learning [13]. This movement encourages students to take greater responsibility for their own learning [14], facilitating greater development of the “lifelong learner” or trainee which is a key aim of many medical education curricula [15]. The effectiveness of this teaching approach has been demonstrated through systematic review and meta-analyses [16].

However, although a statistically significant improvement in question performance was found after the introduction of the new teaching method, it was not possible to run a contemporaneous control group exposed to the previous teaching methods. In addition, whilst the questions were structured very similarly, with similar questions asked, it was not possible to ask the exact same question for 3 years running.

Similarly, one assumption of classical test theory is that items within a set are unidimensional, i.e. that they are all indicative of the same underlying characteristic. However, it could be argued that the skills required to answer a question on the analysis of routine herd-level fertility may be different to those required to answer a question on individual farm animal medicine, which constitutes many of the questions in the rest of the same examination. Interestingly, although an improvement in the R-squared value was noted in the year following the introduction of the improved teaching method (0.11), this question was still on the lower range of values for other questions in the examination (Range: 0.11–0.47).

## Conclusion

In conclusion, the combination of qualitative focus group student feedback and quantitative data analysis of examination performance represent useful methods for identifying problems associated with specific aspects of teaching farm animal veterinary teaching, highlighting potential ways to improve that teaching, and to monitor the impact of those changes on student performance.

## Data Availability

Data are not available consistent with the conditions of the ethical exemption.

## Abbreviations

AI: Artificial Insemination
CTT: Classical Test Theory
FACS: Farm Animal Clinical Studies
KPI: Key Performance Indicators
UCD: University College Dublin
